# Patient Experience of Emergency Laparotomy: A Mixed Methods Study (The PEEL‐2 Study)

**DOI:** 10.1002/wjs.70342

**Published:** 2026-04-09

**Authors:** Louise M. Silva, Sarah A. Mohammed, Paula J. Strong, Alina Dietrich, Tessa Watts, Jonathan Bisson, Jared Torkington, Julie A. Cornish

**Affiliations:** ^1^ University Hospital of Wales Cardiff UK; ^2^ Cardiff University Cardiff UK

## Abstract

**Background:**

Emergency laparotomy (EmLap) is a high‐risk surgery for acute abdominal conditions. This study uses a retrospective mixed‐methods approach to explore quality of life and patient‐reported outcomes and experiences (PROMs and PREMs) among EmLap survivors.

**Methods:**

Patients who underwent EmLap from 2016–2019 at a tertiary hospital were surveyed, with demographic and clinical data collected from institutional National Emergency Laparotomy Audit (NELA) databases. Outcomes included QoL (EQ‐5D‐5L), employment, sexual function, incisional hernias, peri‐operative anxiety, body image, and overall patient experience. The themes explored in the questionnaire were developed based on findings from previous studies of EmLap survivorship. Thematic analysis was conducted for qualitative responses, alongside statistical analysis for quantitative data.

**Results:**

A total of 725 eligible patients were identified, and 310 responses were returned (42.8% response rate). Mean length of follow up was 33 months (range 6–54 months). Regression analysis confirmed QoL was associated with higher socioeconomic status (coefficient 0.047, *p* < 0.001), reduced BMI (coefficient −0.009, *p* < 0.05) and reduced ASA (coefficient −0.070, *p* < 0.05). Nearly half of respondents were retired at the time of surgery; among those who were employed, 40.5% experienced changes in employment which included earlier retirement. Recovery of sexual function was delayed, with 11.3% of patients reporting they had not resumed sexual activity post‐surgery. Incisional hernia was reported by 38.9% of respondents, and 34.0% expressed dissatisfaction with body image. Free‐text responses revealed unmet needs for mental health support (22.6%), dietary guidance (10.3%), and physiotherapy (15.8%).

**Conclusion:**

These findings highlight the physical, psychological, and socioeconomic burden faced by patients after EmLap, and support the need for tailored post‐operative support systems to be integrated into post‐operative care.

## Summary

What does this paper add to the literature?

- Presents the first large‐scale, mixed‐methods study with long‐term follow‐up assessing quality of life (QoL), PROMs, and PREMs in emergency laparotomy (EmLap) survivors.
- Identifies key long‐term sequelae including incisional hernia, impaired return to work, disrupted sexual function, and negative body image.
- Highlights significant unmet needs in post‐operative care, particularly in mental health, dietary support, and physiotherapy.
- Observes an association between higher socioeconomic status with improved QoL and reduced risk of incisional hernia, suggesting socioeconomic factors may influence recovery.
- Suggests a potential link between pre‐existing depression and incisional hernia development, which may warrant further exploration.
- Supports the case for integrated, multidisciplinary rehabilitation pathways tailored to EmLap patients, drawing on models used in cancer or cardiac care.

## Graphical abstract

Key Findings from the PEEL2 Study: EmLap survivors experience lasting effects on quality of life, employment, and sexual function, and want more support during their recovery.

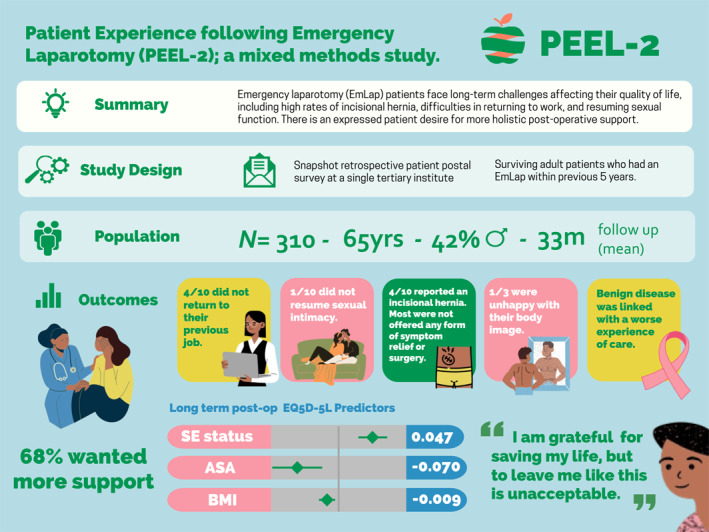

## 1 Introduction

Emergency laparotomy (EmLap) is a surgical procedure performed to address a range of acute abdominal conditions, including bowel obstruction, perforation, ischemia, and intra‐abdominal hemorrhage. While the underlying pathology and technical approach may vary, the unifying factor is the urgent need to surgically access the abdomen without delay.

Inevitably, this level of urgency carries significant risk. According to the latest National Emergency Laparotomy Audit (NELA), the 30‐day mortality rate stands at 9.0% [1]. Traditionally, mortality has been the primary benchmark for determining surgical success. However, as survivorship improves, patient priorities are shifting toward Quality of Life (QoL), recognizing that survival alone does not guarantee a meaningful recovery, nor wellbeing [2]. Gaining a clear understanding of changes in QoL following EmLap is crucial for guiding shared decision‐making in the context of such high‐risk surgery.

The World Health Organization (WHO) defines QoL as an individual's perception of their position in life, shaped by their cultural, societal, and personal contexts, as well as their goals, expectations, standards, and concerns [3]. However, capturing and quantifying the complexity within this definition is inherently challenging.

Psychosocial impacts are often intricate, long‐lasting, and intangible, stretching well beyond the scope of a standard post‐op clinic review. Current EmLap care pathways lack the depth needed to both appreciate and address changes in QoL. Whilst Patient Reported Outcome Measures (PROMs) and Patient Reported Experience Measures (PREMs) are well accepted to quantify changes in QoL following scheduled surgery [4, 5, 6], their application in an emergency setting is more problematic [7]. Consequently, the understanding of QoL following EmLap remains comparatively limited.

A recent systematic review revealed substantial variation in reported QoL following EmLap [8]. The findings allude to an age‐specific, two‐tailed pattern, with younger patients often experiencing improvements, while older patients tend to face impairments [8]. However, the review also highlighted notable gaps in the literature, including inconsistent use of PROM instruments and variability in follow‐up durations, both of which weaken the reliability of conclusions [8].

Much of the existing research consists of feasibility studies [9, 10, 11, 12], short‐term follow‐up 10, 11, 13, 10, 11, 13, or single‐method approaches [2, 14, 15, 16]. This leaves a significant gap in understanding the depth and context of QoL following EmLap. At present, no mixed‐method studies with long‐term follow‐up exist, creating barriers to both informed shared decision‐making and the development of effective interventions to improve holistic recovery.

## 2 Aims

This study aims to describe long term QoL following EmLap using a mixed methods approach via patient survey.

## 3 Methods

### 3.1 Study Design

This is a retrospective mixed methods snapshot patient survey, involving all surviving patients who underwent an EmLap within a 5‐year period at a tertiary university hospital. Inclusion criteria consisted of patients who had an EmLap meeting NELA criteria between 1st January 2016 – 31st December 2019. Exclusion criteria included mortality at the time of study; a known capacity issue preventing survey completion; or inability to read/write in English. Patients with more than one EmLap admission during the study period were included once; repeat admissions were not included nor analyzed.

### 3.2 Outcomes

Outcomes included QoL as measured by EQ‐5D‐5L, the rate of return to employment, patient‐reported incidence of incisional hernia (IH), the rate of return to sexual function, patient recollection of peri‐operative anxiety, change in body image perception, and patient experience of EmLap care.

### 3.3 Survey Development

Currently no validated outcome tool exists to comprehensively describe EmLap recovery. While the EQ‐5D‐5L is widely used to assess QoL, its generic nature often overlooks key aspects of surgical recovery [17]. To address this limitation, additional questions were developed to ensure a more thorough evaluation of outcomes specific to EmLap surgery. Previous focus group research at our institute has identified key themes of EmLap survivorship; return to work; incisional hernia; sexual function; overall quality of life; peri‐operative anxiety; body image; overall experience of care; and sources of support [18]. These themes informed the development of an 18‐point questionnaire comprising of both open and closed questions (see supplementary material). The questionnaire was developed by the research team in collaboration with a specially convened multi‐disciplinary advisory group, comprising representatives from general surgery, nursing, anesthetics, peri‐operative physician care, primary care, and mental health. All questionnaire responses were captured and analyzed as categorical data.

### 3.4 Study Procedure

This study was reviewed by the local R&D department, which determined that ethical approval was not required. As a result, it was granted approval as a service evaluation. Surveys were distributed with a pre‐paid, pre‐addressed return envelope and a covering letter detailing the project's purpose. The return of a completed questionnaire was taken as implied informed consent. Surveys were distributed to EmLap survivors 6–54 months post‐event, ensuring a broad sample while avoiding hyperacute responses.

### 3.5 Covariables

Demographic and clinical data were obtained by matching patients with electronic records and our local NELA database. Socioeconomic status was assessed using the Welsh Index of Multiple Deprivation (WIMD) tool [19]. WIMD ranks all small areas in Wales from 1 (lowest socioeconomic status) to 1909 (highest socioeconomic status). It is a National Statistic produced by statisticians at the Welsh Government. Small areas are Census geographies called Lower‐layer Super Output Areas (LSOAs).

The full index is updated every 4–5 years and Indices are determined by 8 factors including income, employment, health, education, access to services, housing, community safety, physical environment for that specific area [19].

### 3.6 Analysis

#### 3.6.1 Qualitative Analysis

Free‐text comments were analyzed using inductive thematic analysis, following the approach outlined by Braun and Clarke [20]. Two researchers (LS and TW) identified the major themes, and the comments were then coded (LS) to determine the frequency of themes and subthemes. To reduce subjectivity and potential bias, 25% of the responses were randomly selected for double‐coding by a second researcher (SM). Any discrepancies or disagreements were resolved collaboratively through discussion, with adjustments made as necessary.

#### 3.6.2 Quantitative Analysis

Data was analyzed using SPSS version 27.0 (SPSS Inc., Chicago, IL, USA). For descriptive analysis, non‐parametric data were expressed as the median and interquartile range (IQR), while parametric data were expressed as the mean and standard error of the mean. Categorical data were presented as percentages or frequencies.

For inferential analysis, the distribution of clinical variables between respondents versus non‐respondents, and positive versus negative experiences, was assessed using Pearson's chi‐squared (χ^2^) test for categorical variables, and the independent *t*‐test for continuous variables.

Univariable logistic regression analysis was employed to quantify the strength of associations between independent clinical variables, and patient‐reported outcome. Independent reference variables were determined based on the distribution of the mean (e.g., age and socioeconomic status) or the “most disease‐free state” (e.g., ASA 1 vs. others, and non‐malignant disease vs. malignancy), unless otherwise specified. Data were presented as odds ratios (OR), 95% confidence intervals (CI), and levels of significance.

Stepwise multiple linear regression was used to identify factors significantly influencing QoL. The EQ‐5D‐5L responses were weighted using the population value set developed by Devlin et al. for England [21]. Each EQ‐5D‐5L response was mapped to its corresponding index value, and the EQ‐5D‐5L indices were used as the dependent variable for regression analysis. A maximum of 10 independent variables were selected based on theoretical relevance and statistical criteria. Variables demonstrating multicollinearity were excluded using Variance Inflation Factor (VIF) thresholds. All statistical tests were two‐sided, and statistical significance was set at *α* = 0.05.

## 4 Results

### 4.1 Sample Characteristics

**FIGURE 1:**
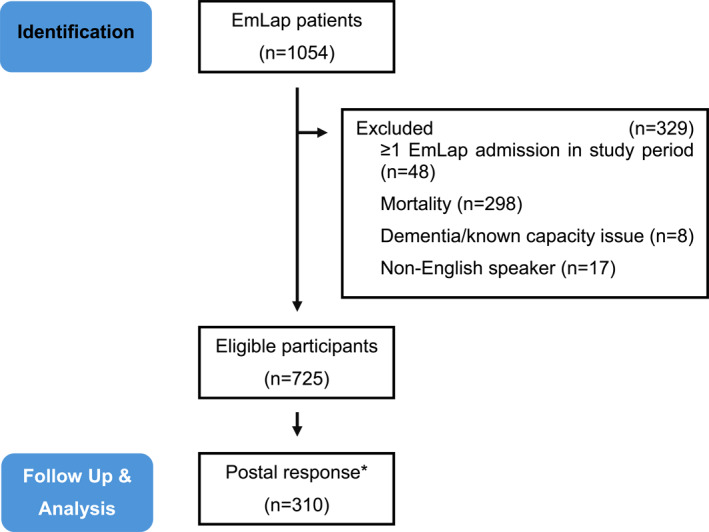
Flow chart of study procedures. * refers to pooled responses (local questionnaire & EQ‐5D‐5L).

A total of 1054 EmLap records were documented in our local database, of which 725 patients met the eligibility criteria for the study. Out of these, 310 responses were received, yielding a response rate of 42.8% (Figure 1). The median age of respondents was 65 years (IQR 54–73), and 41.9% were male. No significant differences were observed between respondents and non‐respondents in terms of sex, BMI, ASA, or cancer status (Table 1). Mean length of follow up was 33 months (range 6–54 months).

**TABLE 1:** Characteristics of Respondents and Non‐respondents.

Variable	Total eligible		Respondents		Non‐respondents		Pearson's x^2^	*p*
	*N*	% of total	*n*	% of respondents	*n*	% of non‐respondents		
Age							50.293	0.000*
Median (IQR)	61 (24–73)		65 (54–73)		58 (42–73)			
18 – 40 years	125	17.2	31	10.0	94	22.7		
41 – 60 years	205	28.3	77	24.8	128	30.8		
61 – 80 years	313	43.2	178	57.4	135	32.5		
81 – 100 years	82	11.3	24	7.7	58	14.0		
Sex							2.389	0.122
Male	328	45.2	130	41.9	198	47.7		
Female	397	54.8	180	58.1	217	52.3		
BMI							4.203	0.379
Median	26.5 (23.2–31.9)		26.6 (23.9–31.5)		26.5 (23.1–32.4)			
Underweight	13	2.6	5	2.3	8	2.9		
Healthy BMI	172	34.8	73	33.2	99	36.1		
Overweight	140	28.3	72	32.3	68	24.8		
Obese	92	18.6	40	18.2	52	19.0		
Morbidly obese	77	15.6	30	13.6	47	17.2		
Socioeconomic status							17.507	0.002*
Lowest socioeconomic status	173	25.2	59	19.9	114	29.3		
2^nd^ quintile	102	14.9	39	13.1	63	16.2		
3^rd^ quintile	91	13.3	35	11.8	56	14.4		
4^th^ quintile	127	18.5	60	20.2	67	17.2		
Highest socioeconomic status	193	28.1	104	35.0	89	22.9		
Education level^a^								
Level 1–2	—	—	94	35.1	—	—		
Level 3	—	—	91	34.0	—	—		
Level 4 – 6	—	—	40	14.9	—	—		
Level 7	—	—	65	13.1	—	—		
Level 8	—	—	8	3.0	—	—		
Employment status^a^					—	—		
Full time	—	—	61	21.1	—	—		
Part time	—	—	28	9.7	—	—		
Self employed	—	—	21	7.3	—	—		
Retired	—	—	140	48.4	—	—		
Unemployed	—	—	9	13.5	—	—		
ASA							2.711	0.607
1	85	11.8	34	11.0	51	12.3		
2	289	40.0	125	40.5	164	39.6		
3	267	36.9	116	37.5	151	36.5		
4	75	10.4	33	10.7	42	10.1		
5	7	1.0	1	0.3	6	1.4		
Comorbidities								
Diabetes	45	6.2	21	6.8	24	5.8	0.325	0.569
Respiratory disease	24	3.4	11	3.6	13	3.3	0.052	0.820
Cardiac disease	9	1.3	5	1.6	4	1.0	0.533	0.465
CKD3	17	2.4	9	2.9	8	2.0	0.623	0.430
Previous mental health diagnosis								
Any diagnosis	275	40.0	105	34.8	170	44.2	6.213	0.013
Depression	189	27.5	82	27.2	107	27.8	0.035	0.852
Anxiety	110	16.0	38	12.6	72	18.7	4.711	0.030*
Chronic pain	65	9.4	43	14.2	22	57.9	14.243	0.000*
Risk prediction							0.047	0.828
Low, < 5%	314	51.4	137	51.9	177	51.0		
High, > 5%	297	48.6	127	48.1	170	49.0		
Surgical procedure								
Adhesiolysis	175	24.1	82	26.5	93	22.4	1.583	0.208
Small bowel resection	74	10.2	26	8.4	48	11.6	1.957	0.162
Right hemicolectomy	95	13.1	42	13.5	53	12.8	0.094	0.759
Hartmann's	97	13.4	55	17.7	42	10.1	8.894	0.003*
Duodenal ulcer repair	44	6.1	11	3.4	33	8.0	6.036	0.014*
Cancer diagnosis	116	16.2	56	18.2	60	14.6	1.708	0.191
New stoma	276	61.6	140	45.6	136	33.8	10.308	0.001*

^a^ Obtained from survey responses.

### 4.2 Quality of Life

**FIGURE 2:**
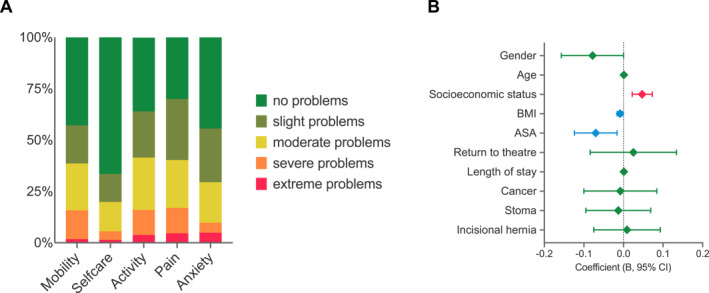
EQ5D‐5L responses. [A] Bar chart demonstrating distribution of responses across domains. [B] Forest plot demonstrating predictors influencing EQ5D‐5L indices.

A total of 279 respondents provided complete EQ‐5D‐5L responses. The distribution of responses across all domains is shown in Figure 2a, with a median EQ‐5D‐5L index of 0.78 (IQR: 0.57–0.92). Regression analysis identified significant predictors of EQ‐5D‐5L, summarized in Table 2. Higher socioeconomic status demonstrated a positive association with EQ‐5D‐5L, with an unstandardized coefficient of 0.047, indicating that each unit increase in socioeconomic status corresponded to a 0.047 increase in index value (*p* < 0.001). In contrast, ASA classification showed a significant negative association (*p* < 0.05), as reflected by a standardised coefficient of −0.070. Similarly, BMI exhibited a smaller negative effect with an unstandardized coefficient of −0.009 (*p* < 0.05). Conversely, cancer diagnosis, presence of a stoma, and incisional hernia did not demonstrate significant relationships with EQ‐5D‐5L and had minimal contributions to the model (Figure 2b). In addition, 286 respondents completed EQ‐5D‐VAS assessments. The median EQ‐5D‐VAS score was 70 (IQR: 50–80).

**TABLE 2:** Coefficients for EQ‐5D‐5L indices.

Predictor	Unstandardized coefficient (B)	Standard error (SE B)	Standardised coefficient (Beta)	t‐statistic	*p*‐value	95% confidence interval (lower, Upper)		VIF
(Constant)*	1.036	0.186	—	5.566	< 0.001	0.669	1.404	—
Sex	−0.078	0.040	−0.141	−1.962	0.051	−0.157	0.000	1.094
Age	0.001	0.001	0.040	0.543	0.588	−0.002	0.003	1.129
Higher socioeconomic status (quintile)*	0.047	0.013	0.265	3.636	< 0.001	0.022	0.073	1.122
BMI*	−0.009	0.003	−0.203	−2.663	0.008	−0.016	−0.002	1.227
ASA*	−0.070	0.027	−0.201	−2.574	0.011	−0.124	−0.016	1.278
Return to theater	0.025	0.055	0.035	0.455	0.650	−0.084	0.134	1.246
Length of stay	0.001	0.001	0.057	0.669	0.505	−0.002	0.003	1.549
Cancer	−0.008	0.047	−0.013	−0.179	0.858	−0.100	0.084	1.076
Stoma	−0.013	0.042	−0.024	−0.312	0.756	−0.095	0.069	1.245
Incisional hernia	0.009	0.043	0.017	0.221	0.825	−0.075	0.093	1.248

### 4.3 Employment and Return to Work

Nearly half of respondents (48.4%) were retired at the time of undergoing EmLap. Among those who were employed, 38.1% returned to work within 1–3 months. However, 40.5% were unable to resume their previous job, instead becoming unemployed, reducing their working hours, or transitioning to different roles. Respondents were more likely to require job changes if they were female (OR 2.858, *p* < 0.05), had a higher ASA score (ASA 3 OR 5.000, *p* < 0.05), developed a new stoma (OR 4.006, *p* < 0.001), or experienced an incisional hernia (OR 4.228, *p* < 0.001; Table 3).

**TABLE 3:** Univariable Logistical Regression for Poor Patient Reported Outcomes: Incisional Hernia, Peri‐operative Anxiety, Unsatisfied Body Image, Failure to Resume Sexual Function, and Change in Employment.

	Patient reported incisional hernia			Patient reported peri‐op anxiety			Long term unsatisfied body image			Failure to resume sexual function			Change in employment		
	OR	CI	*p*	OR	CI	*p*	OR	CI	*p*	OR	CI	*p*	OR	CI	*p*
Age			0.197			0.169			0.082			0.003*			0.454
18 – 40 years	0.629	0.279–1.419		0.0590	0.278–1.251		1.975	0.917–4252		0.840	0.167–4.213		1.491	0.524–4.244	
41 – 60 years	0.886	0.513–1.531		0.856	0.481–1.525		1.547	0.881–2.718		1.424	0.506–4.008		1.010	0.415–2.456	
61 – 80 years	1.0	Reference		1.0	Reference		1.0	Reference		1.0	Reference		1.0	Reference	
81 – 100 years	0.495	0.170–1.441		0.629	0.300–1.320		0.846	0.288–2.486		10.500	2.197–50.177		na	na	
Sex			0.184			0.614			0.049*			0.615			0.009*
Male	1.0	Reference		1.0	Reference		1.0	Reference		1.0	Reference		1.0	Reference	
Female	1.389	0.856–2.254		0.886	0.552–1.421		1.659	1.002–2.746		0.795	0.326–1.939		2.858	1.306–6.257	
BMI			0.011*			0.121			0.016*			0.470			0.283
Underweight	na	na		1.091	0.141–8.420		na	na		2.467	0.213–28.535		4.004	0.319–50.229	
Healthy BMI	1.0	Reference		1.0	Reference		1.0	Reference		1.0	Reference		1.0	Referene	
Overweight	1.887	0.934–3.812		1.374	0.637–2.961		2.488	1.183–5.234		1.233	0.361–4.219		1.000	0.311–3.210	
Obese	1.400	0.600–3.265		0.800	0.303–2.109		2.590	1.087–6.174		0.617	0.111–3.438		2.101	0.457–8.746	
Morbidly obese	3.345	1.320–8.479		2.182	0.813–5.856		3.662	1.417–9.462		0.925	0.162–5.278		2.101	0.457–8.746	
Socioeconomic status			0.008*			0.014*			0.040*						
Lowest socioeconomic status	0.711	0.298–1.695		1.555	0.679–3.562		1.360	0.561–3.300		na	na		0.673	0.175–2.588	
2^nd^ quintile	0.504	0.188–1.353		2.090	0.819–5.339		2.821	1.047–7.599		na	na		0.735	0.157–3.434	
3^rd^ quintile	1.0	Reference		1.0			1.0	—		na	na		1.0		
4^th^ quintile	0.388	0.161–0.932		3.264	1.273–8.369		0.760	0.308–1.874		na	na		0.429	0.099–1.857	
Highest socioeconomic status	0.330	0.146–0.744		2.004	0.891–4.506		0.611	0.263–1.418		na	na		0.426	0.118–1.560	
ASA			0.006*			0.335			0.200			0.153			0.024*
1	1.0	Reference		1.0	Reference		1.0	Reference		1.0	Reference		1.0		
2	4.732	1.553–14.419		0.819	0.339–1.974		1.382	0.583–3.276		2.842	0.342–23.591		3.548	0.916–13.752	
3	4.637	1.510–14.244		0.652	0.273–1.556		0.909	0.374–2.208		4.632	0.566–37.896		5.000	1.231–20.301	
4	2.632	0.689–10.053		0.900	0.344–2.351		2.022	0.688–5.941		1.600	0.093–27.547		3.333	0.378–29.389	
Mental health diagnosis															
Any diagnosis	0.763	0.465–1.252	0.284	1.261	0.739–2.152	0.395	3.164	1.888–5.305	< 0.001*	1.267	0.512–3.140	0.609	1.746	0.793–3.843	0.166
Depression	1.755	1.038–2.967	0.036*	1.461	0.777–2.747	0.239	3.536	2.047–6.110	< 0.001*	1.503	0.591–3.820	0.392	1.905	0.820–4.424	0.134
Anxiety	0.929	0.459–1.880	0.0837	1.061	0.520–2.164	0.872	1.317	0.637–2.722	0.547	0.314	0.040–2.452	0.269	2.408	0.638–9.090	0.195
Risk prediction			0.179			0.511			0.078			0.855			0.084
Low, < 5%	1.0	Reference		1.0	Reference		1.0	Reference		1.0	Reference		1.0	Reference	
High, > 5%	1.420	0.852–2.366		0.843	0.506–1.406		1.611	0.949–2.736		0.916	0.358–2.346		2.105	0.904–4.902	
Surgical procedure^‡^															
Adhesiolysis	0.370	0.205–0.670	< 0.001*	0.839	0.479–1.468	0.538	0.772	0.440–1.357	0.369	0.591	0190–1.837	0.363	0.347	0.155–0.965	0.042
Small bowel resection	0.667	0.265–1.676	0.309	1.147	0.541–2.429	0.720	0.967	0.399–2.345	0.940	1.464	0.302–7.082	0.636	0.886	0.201–3.906	0.873
Right hemicolectomy	0.441	0.208–0.938	0.033	1.147	0.541–2.429	0.720	0.583	0.273–1.245	0.163	0.812	0.225–2.930	0.751	0.460	0.117–1.804	0.460
Hartmann's	3.305	1.763–6.195	< 0.001*	1.318	0.408–4.257	0.645	2.383	1.289–4.408	0.006*	1.580	0.537–4.644	0.406	2.085	0.785–5.541	0.141
Duodenal ulcer repair	0.382	0.080–1.832	0.229	1.354	0.701–2.614	0.367	0.826	0.209–3.265	0.785	Na			0.358	0.039–3.312	0.365
Approach			0.035*			0.036*			0.028*			0.841			0.358
Open	2.457	1.067–5.657		2.177	1.051–4.508		2.365	0.973–5.553		1.142	0.312–4.181		1.304	0.465–3.656	
Laparoscopic	1.0	Reference		1.0	Reference		1.0	Reference		1.0	Reference		1.0	Reference	
Lap converted	1.446	0.477–4.389		2.000	0.710–5.630		8.286	1.255–54.705		1.083	0.163–7.203		0.429	0.092–31.924	
Contamination	1.759	1.087–2.847	0.021*	0.770	0.476–1.246	0.286	2.065	1.257–3.393	0.004*	1.009	0.409–2.488	0.985	1.498	0.701–3.200	0.297
Critical care	0.766	0.465–1.264	0.298	1.140	0.700–1.857	0.599	1.147	0.687–1.915	0.600	1.896	0.760–4.732	0.170	1.733	0.745–4.035	0.202
Return to theater			0.010*			0.864			0.723			0.016*			0.169
No return	1.0	Reference		1.0	Reference		1.0	Reference		1.0	Reference		1.0	Reference	
Unplanned return	3.747	1.379–10.178		0.924	0.372–2.296		1.160	0.441–3.050		5.111	1.363–19.162		2.838	0.641–12.564	
Cancer diagnosis			0.276			0.163			0.792			0.197			0.555
Benign disease	1.0	Reference		1.0	Reference		1.0	Reference		1.0	Reference		1.0	Reference	
New Ca diagnosis	1.142	0.577–2.261		1.717	0.804–3.663		1.044	0.517–2.108		0.259	0.033–2.014		1.286	0.401–4.122	
Previous Ca diagnosis	2.250	0.523–9.673		0.736	0.298–1.820		0.831	0.209–3.298		1.400	0.156–12.604		0.500	0.050–4.988	
IBD diagnosis			0.160			0.270			0.412			0.009*			0.171
No IBD	1.0	Reference		1.0	Reference		1.0	Reference		1.0	Reference		1.0	Reference	
New diagnosis	2.078	0.750–5.757		0.453	0.111–1.852		0.576	0.155–2.148		na	na		4.974	0.499–49.544	
Previous diagnosis	0.718	2.150–2.395		0.776	0.253–2.377		1.281	0.442–3.716		5.00	1.502–16.643		2.11	0.469–10.416	
New stoma	3.829	2.319–6.320	< 0.001*	1.104	0.660–1.829	0.702	2.253	1.370–3.706	< 0.001*	4.906	1.729–13.917	0.003*	4.006	1.779–90.52	< 0.001*
IH Reported	—	—	—	—	—		2.843	1.702–4.749	< 0.001*	3.250	1.273–8.298	0.014*	4.228	1.852–9.653	< 0.001*

^‡^ Reference is all other procedures grouped.

Additionally, 23.0% of respondents reported that EmLap served as a catalyst for taking early retirement. Free‐text responses examining why respondents changed their employment identified three major themes: deconditioning and fatigue, employer dismissal, and delays to reconstructive surgery. Many respondents expressed the belief that the EmLap “episode” was incomplete until reconstructive surgery had been performed.

### 4.4 Sexual Function

Of the respondents, 194 (63.4%) were sexually active at the time of their EmLap. The majority (41.2%) took more than 6 months to resume any form of intimacy post‐operatively, while 11.3% reported that they had not resumed sexual activity at all (mean follow‐up: 33 months). Respondents were significantly less likely to *resume* sexual activity if they experienced an unplanned return to theater (OR 5.111, *p* < 0.05), had a pre‐existing diagnosis of Inflammatory Bowel Disease (IBD; OR 5.00, *p* < 0.05, Table 3), received a new stoma (OR 4.906, *p* < 0.05), or were over 80 years old at the time of EmLap (OR 10.500, *p* < 0.05).

### 4.5 Patient Reported Incidence of Incisional Hernia

A total of 38.9% of respondents reported developing an incisional hernia (IH) following EmLap. Respondents were significantly more likely to report IH if they were morbidly obese (OR 3.345, *p* < 0.05), had a higher ASA (ASA 2 OR 4.732, *p* < 0.05), or had a past medical history of depression (OR 1.755, *p* < 0.05; Table 3). Higher socioeconomic status appeared to be a protective factor, with respondents in the fifth socioeconomic quintile having a lower risk of reporting IH (OR 0.380, *p* < 0.05).

Respondents who underwent an open (*n* = 214) rather than a laparoscopic (*n* = 34) EmLap were also more likely to report IH (OR 2.457, *p* < 0.05). Those who had undergone a Hartmann's procedure were at the highest risk of IH (OR 3.305, *p* < 0.001), which may explain why respondents with a new stoma (any type) were also at high risk (OR 3.829, *p* < 0.001). Additionally, significant contamination (OR 1.759, *p* < 0.05) or an unplanned return to theater (OR 3.747, *p* < 0.05) further increased the risk of reporting IH.

Only 32.5% of respondents who reported IH indicated they were offered corrective surgery. The majority (36.9%) reported not being offered any form of treatment or symptom relief, while the remaining 30.6% were provided with external support. Notably, respondents who were obese at the time of the original surgery were less likely to be offered surgical repair (OR 0.250, *p* < 0.05).

### 4.6 Body Image

A total of 34.0% of respondents reported feeling unsatisfied or very unsatisfied with their present‐day body image. Analysis revealed no significant difference in patient perceptions of body image between the time of surgery and present day (χ^2^ 4.391, *p* = 0.36). Respondents who remained “unsatisfied” or “very unsatisfied” with their body image were more likely to be female (OR 1.659, *p* < 0.05), overweight or obese (OR 2.488, *p* < 0.05), from a lower socioeconomic background (second Quintile OR 2.821, *p* < 0.05), or have a previous mental health diagnosis (OR 3.164, *p* < 0.001), particularly depression (OR 3.536, *p* < 0.001; Table 3).

Respondents who had undergone an open procedure did not show significantly worse body image outcomes compared to those who had laparoscopic surgery (OR 2.365, *p* = 0.058). However, individuals who underwent laparoscopic surgery, later converted to open (*n* = 36), had markedly higher dissatisfaction with their body image (OR 8.265, *p* < 0.05). Additionally, factors such as Hartmann's procedure, a new stoma (of any type), and reported IH were significant contributors to body image dissatisfaction (OR 2.383, *p* < 0.006; OR 2.253, *p* < 0.001; OR 2.843, *p* < 0.001, respectively).

### 4.7 Post‐Operative Anxiety

A total of 48.2% of respondents recalled experiencing significant peri‐operative anxiety during their inpatient admission. Respondents with higher socioeconomic status (fourth Quintile OR 3.264, *p* < 0.05; Table 3) and those who underwent an open procedure (OR 2.177, *p* < 0.05) were more likely to report peri‐operative anxiety. However, the study did not find a significant difference in reported peri‐operative anxiety between respondents with malignant versus benign disease (OR 1.717, *p* = 0.163).

### 4.8 Patient Reported Experience

A total of 185 respondents provided a free‐text response describing their experience of care. Respondents with benign disease were significantly more likely to report negative experiences compared to those with malignant disease (Pearson's χ^2^ 6.679, *p* < 0.05).

The analysis identified major themes within patient feedback, including lack of holistic support, unmet recovery expectations, communication failures, feelings of abandonment post inpatient stay, and diminished confidence in healthcare services. The most frequently reported unmet need was surgical aftercare (25.2%). Additionally, 22.6% of respondents indicated a need for more mental health support, 10.3% felt dietician involvement was insufficient, and 15.8% expressed a lack of physiotherapy support. Notably, 12.3% of respondents reported being unaware of the details of their procedure, with two respondents uncertain whether a malignancy had been removed. There was a strong desire for a post‐discharge “debrief” outside the acute clinical setting to clarify the sequence of events.

Further concerns included dissatisfaction with delays to corrective surgery (11.7%) and difficulties accessing aftercare services (10.3%), which frequently involved unreturned calls. Patients expressed frustrations with comments such as: “*Every time I ask for help – no one wants to help. It feels like ‘we saved your life – we don't want anything more to do with you, you're not our problem anymore*.’” *Female, 60 years, NQ57*.

There was also anger and regret, exemplified by statements like: “*I* *am grateful for saving my life, but to leave me like this is unacceptable.*” *Female, 65 years, NQ25.* Further examples of quotes are provided in Table S1 (supplementary material).

In terms of desired support, 68.6% of respondents indicated they needed additional help. Specifically, 88.1% stated they would have benefitted from a specialist nurse, 24.5% expressed interest in an EmLap‐specific patient group, and 13.5% preferred support through an online platform or app.

## 5 Discussion

This study presents the first large‐scale, mixed‐methods evaluation of long‐term outcomes following EmLap, capturing both patient‐reported outcomes (PROMs) and experiences (PREMs). Key findings include a high prevalence of incisional hernia (38.9%), delayed or disrupted return to work (40.5%), and persistent challenges with sexual function (11.3% not resumed). Quality of life (QoL) was significantly associated with socioeconomic status, ASA grade, and BMI. Qualitative data revealed widespread dissatisfaction with post‐operative support, particularly in mental health, dietary guidance, and surgical aftercare. Notably, patients with benign disease reported poorer care experiences than those with malignancy.

Our findings align with previous studies reporting high morbidity and psychosocial burden after EmLap, but this study extends the literature by offering long‐term follow‐up and integrating qualitative insights. The observed association between higher socioeconomic status and improved QoL reflects broader evidence linking socioeconomic factors to health outcomes [22]. This study is also the first to suggest a potential protective effect of higher socioeconomic status against the development of incisional hernia in this cohort. Patients from lower socioeconomic status appeared to be at increased risk—possibly due to a clustering of risk factors such as smoking [23], higher ASA scores, and more severe illness at presentation, with greater levels of contamination [24]. While the impact of hernias upon mental health is well documented [25], our findings raise the possibility that pre‐existing depression may also increase hernia risk—an area warranting further exploration.

Incisional hernia had a clear detrimental impact on patients' lives, affecting body image, sexual function, and return to work. Patients with obesity were notably less likely to be offered treatment compared to those with lower BMI, though the reasons remain unclear. This finding is limited by reliance on self‐reporting, without corroborating clinical notes to confirm whether surgical discussions or weight management strategies occurred. While obesity is often considered a risk factor for recurrence, emerging evidence—including the “obesity paradox” [26] and similar morbidity rates in lower obesity classes—challenges this assumption [27]. Reports of implicit weight stigma in surgical settings [28, 29, 30] further raise the possibility that such conversations may be avoided due to assumptions, discomfort, or bias.

Employment disruptions were notably higher than those reported in earlier UK studies, likely due to the extended follow‐up period [31]. Sex disparities were also evident, with women more likely to leave the workforce, which may reflect both health‐related and underlying societal trends [32].

Sexual function and body image were also significantly affected, particularly among patients with stomas and incisional hernias. These findings are consistent with prior research in colorectal and IBD populations, but remain underrepresented in EmLap literature [33, 34].

Qualitative responses were strongly emotive, revealing the aftermath patients experience following EmLap — not only in relation to changes in physical health but also in navigating healthcare services. A clear desire for better information, emotional support, and structured follow‐up suggests that current aftercare pathways are not adequately meeting patient needs. Although these findings reflect a single site and are not generalizable, they underscore the absence of post‐operative standards: unlike the well‐established benchmarks for pre‐operative care, there are no equivalent for longer term post EmLap care. These insights emphasize the importance of patient perspectives and align with ongoing efforts within NELA to strengthen survivorship and recovery support [35].

### 5.1 Strengths and Limitations

A major strength of this study is its mixed‐methods design, enabling a comprehensive understanding of both measurable outcomes and lived experiences. The large sample size and extended follow‐up period provide valuable insights into long‐term recovery.

Limitations include the retrospective, single‐site design, which may introduce recall bias and restrict generalizability. Reliance on patient‐reported outcomes adds depth to understanding patient perspectives but is limited in certain contexts, such as incisional hernia, where accuracy is uncertain without objective clinical assessment. Although recruitment ended before the onset of the Covid‐19 pandemic, a very small minority of patients would have had early follow‐up care directly affected by restrictions. More broadly, the pandemic context may have influenced patients' mood and perceptions of healthcare access, and this potential impact is acknowledged as a limitation. The response rate (42.8%) is moderate, with respondents older and more likely to be from higher socioeconomic groups than non‐respondents, which may have skewed results. The absence of a comparator group limits causal inference, and while the EQ‐5D‐5L is validated, it may not fully capture the complexity of EmLap recovery. Finally, the additional survey tool, though informed by prior qualitative work, has not been formally validated.

### 5.2 Clinical and Research Implications

These findings highlight the need to shift EmLap outcomes beyond survival, toward holistic, patient‐centered measures of recovery. Clinically, there is a clear case for developing structured post‐operative support pathways, including access to mental health services, dietary support, and vocational rehabilitation. The disparity in care experiences between patients with benign and malignant disease suggests a need to extend cancer‐style follow‐up models to *all* EmLap survivors.

From a research perspective, the associations between socioeconomic status, mental health, and long‐term surgical outcomes merit further investigation. Future studies should aim to validate EmLap‐specific PROMs, explore causal pathways, and evaluate interventions to improve long‐term recovery. Ongoing prospective work, such as the POLO study, will be critical in addressing some of these gaps [36].

## Author Contributions

**Louise M Silva:** conceptualization, investigation, writing ‐ review and editing, writing ‐ original draft, methodology, visualization, formal analysis, project administration, data curation. **Dietrich Alina:** methodology. **Sarah A Mohammed:** methodology, investigation, project administration, validation. **Paula J Strong:** investigation, methodology, project administration. **Julie A Cornish:** conceptualization, writing ‐ review and editing, supervision. **Torkington Jared:** supervision, writing ‐ review and editing. **Tessa Watts:** methodology, investigation, validation, writing ‐ review and editing, supervision. **Jonathan Bisson:** investigation, writing ‐ review and editing, supervision.

## Funding

The authors have nothing to report.

## Ethics Statement

This study was reviewed by the local R&D department, which determined that ethical approval was not required and was granted approval as a service evaluation.

## Conflicts of Interest

The authors declare no conflicts of interest

## Supporting information


Supporting Information S1

Supporting Information S2

**Table S1**: Major Themes of Patient Experience and Illustrative Quotes.

## Data Availability

The data that support the findings of this study are available on request from the corresponding author. The data are not publicly available due to privacy or ethical restrictions.
