# Supplementary material for: Patient Experience of Emergency Laparotomy: A Mixed Methods Study (The PEEL‐2 Study)
Source: World J Surg. 2026 Apr 9;50(5):1276–88. doi: 10.1002/wjs.70342 (PMC13206577; doi:10.1002/wjs.70342)
Supplement: Supplementary file 1 — Supporting Information S1 [file WJS-50-1276-s002.pdf]

GIG CYMRU NHS WALES
Bwrdd Iechyd Prifysgol Caerdydd a'r Fro
Cardiff and Vale University Health Board

<Study number>

<Name>
<Address>
<Postcode>

Dear <Name>,

We are writing to you because you had an emergency surgery on your abdomen at the University Hospital of Wales within the last 5 years.

We understand that this type of surgery can have a big effect upon a patient's quality of life. Our aim is to learn from your experience. Specifically, how emergency surgery affected your well-being immediately after the procedure, and in the longer term.

As we work to remodel and improve our services, your feedback (good or bad) will be invaluable in helping us to enhance care for future patients.

We would be very grateful if you could complete the questionnaire attached with friends/family, and return in the pre-paid & pre-addressed envelope. All responses are anonymised and you do not have to take part in this survey. If you choose not to take part – it will not affect your future care with us.

We have enclosed:

- EQ5D health questionnaire
- Post-surgery health questionnaire
- Self-addressed envelope (postage paid)

If you would like more information about our service or how we will use the data you are providing, please contact us

Many thanks for taking the time to consider this survey.

Yours Sincerely,

Mrs. Julie Cornish
Consultant Colorectal Surgeon

Mrs. J Cornish,
Consultant Colorectal Surgeon
Cardiff and Vale UHB
University Hospital of Wales
CF14 4XW
