# Supplementary material for: Patient Experience of Emergency Laparotomy: A Mixed Methods Study (The PEEL‐2 Study)
Source: World J Surg. 2026 Apr 9;50(5):1276–88. doi: 10.1002/wjs.70342 (PMC13206577; doi:10.1002/wjs.70342)
Supplement: Supplementary file 2 — Supporting Information S2 [file WJS-50-1276-s001.pdf]

«Study_ID»

1	**Did you experience symptoms of anxiety or depression related to your surgery in the recovery period? (please tick)**	☐ Yes<br>☐ No
2	**Before your operation, did you suffer with…? (please tick)**	☐ Anxiety<br>☐ Depression<br>☐ Chronic pain
3	**If yes to any of the above, were you referred to a specialist for support? (please tick)**	☐ Yes<br>☐ No
4	**How satisfied did you feel about your body image, immediately after your operation? (please tick)**	☐ Very unsatisfied<br>☐ Unsatisfied<br>☐ Neutral<br>☐ Satisfied<br>☐ Very satisfied
5	**How satisfied did you feel about your body image, TODAY? (please tick)**	☐ Very unsatisfied<br>☐ Unsatisfied<br>☐ Neutral<br>☐ Satisfied<br>☐ Very satisfied
6	**When did you return to your normal intimacy/sex after your operation? (please tick)**	☐ Not applicable<br>☐ Less than 1 month<br>☐ 1 – 3 months<br>☐ 3 – 6 months<br>☐ More than 6 months
7	**Have you developed any hernias from your surgery? (please tick)**	☐ Yes<br>☐ No
8	**If yes, how is it being managed? (please tick)**	☐ No treatment<br>☐ External support, e.g. TRUSS or corset<br>☐ Surgery/awaiting surgery<br>☐ Other, please state ___________________
9	**What support did you receive in the community after your surgery? (please tick)**	☐ Family/friends<br>☐ Support group<br>☐ Online resources<br>☐ GP/ Hospital team<br>☐ Stoma nurses

«Study_ID»

		☐ Other, please state
10	**What could we have done better during your time in hospital?**	
11	**What is the highest level of education you have completed? (please tick)**	☐ School leavers<br>☐ GCSEs or equivalent<br>☐ A levels or equivalent<br>☐ Undergraduate programme<br>☐ Postgraduate programme
12	**What was your employment status at the time of your operation? (please tick)**	☐ Unemployed<br>☐ Part-time<br>☐ Full-time<br>☐ Self-employed<br>☐ Retired
13	**If employed, how long after your surgery did you return to work? (please tick)**	☐ Less than 1 month<br>☐ 1-3 months<br>☐ 3-6 months<br>☐ More than 6 months
14	**Have you returned to the same employment? (please tick)**	☐ Yes<br>☐ No
15	**If no, what influenced your decision?**	
16	**If there was specific support available for patients after emergency surgery, would you have wanted to access it? (please tick)**	☐ Yes<br>☐ No
17	**How would you have liked to access it? (please tick all that apply)**	☐ Support group meeting<br>☐ Social media (e.g. Facebook)<br>☐ Individual support (e.g. nurse)
18	**What could we have done better to help your recovery after leaving hospital?**	

Thank you for taking the time to complete this questionnaire.

Please return in the pre-paid and addressed envelope.

Thank you
