# Supplementary material for: Patient Experience of Emergency Laparotomy: A Mixed Methods Study (The PEEL‐2 Study)
Source: World J Surg. 2026 Apr 9;50(5):1276–88. doi: 10.1002/wjs.70342 (PMC13206577; doi:10.1002/wjs.70342)
Supplement: Supplementary file 3 — Table S1: Major Themes of Patient Experience and Illustrative Quotes. [file WJS-50-1276-s003.docx]

*Table S1: Major Themes of Patient Experience and Illustrative Quotes (Supplementary)*

| Major themes | Sub-themes | Illustrative Quotes |
| --- | --- | --- |
| Availability of holistic support | - Mental health support - Dietetics - Physiotherapy - Patient support group - Social services - Stoma nurse - Surgical follow up - Obtaining supplies (e.g. home TPN, adequate analgesia on discharge and wound dressings) | *There was a lack of advice, support and information regarding how to rebuild stomach muscles and core muscles after they had been cut. I looked online but nothing - in the end I went to my friend who is a physiotherapist. He told me that it is assumed that people return to desk jobs or are retired. I am a dance teacher, and it was imperative I had exercises given to me specifically to rebuild core and stomach. I am shocked that most people after abdominal surgery never regain their core muscle strength properly which can lead to so many other problems including back pain and digestive problems and posture*. (Female, 50yrs, NQ94)  *It would have been good to have someone to discuss diet and health/nutrition with - no-one did this! Contact with fellow patients would also have been good to help with the post-op depression/understanding. My life has changed, and I am limited to what I can do - seeing other people with similar problems would help to see how they are. I would have liked more assistance to get back to work - but real assistance, not just pen pushing. Advice on health and nutrition for my condition. Someone who could have put me in contact with specialist, one to one advice - tailor made for the individual* (Female, 47yrs, NQ478)  *I developed a form of PTSD, maybe this could have been picked up earlier. I needed counselling after. The NHS was at least 6 months wait so I went privately, then my GP put me on anti-depressants.* (Male, 62yrs, NQ162)  *I needed to talk to somebody regularly, or to meet up in a group related session, or to have a telephone number to ring if so needed.* (Female, 49yrs, NQ98) |
| Expectations regarding recovery | - Feeling unprepared - Hernia ignorance - Discrepancy of expectations between clinician and patient - Understanding of services | *More than 3 days’ worth of pain killers would have made life easier. A community follow up within the first week of leaving hospital would be highly beneficial.* (Female, 43yrs, NQ82)  *Not being told after surgery that I could get a hernia.* (Male, 52yrs, NQ907)  *I wasn’t sure if “this was normal.”* (Male, 64yrs, NQ28)  *"It felt like here's your bag - goodbye" These surgeons don't have a clue what goes on after. At times I've felt I wished they didn't save my life. I never knew I had Crohns’ - I was fine and a body builder and then I went in with my appendix and came out with a bag.* (Male, 29yrs, NQ34)  *The district nurse should have visited me. I had to go to them and my practice nurse I had to rely on taxi drivers.* (Male, 60yrs, NQ207)  *I feel like "wow you're alive, what am I complaining about?”, but help with body image for regaining myself, would have been helpful. The experience itself was worse for my family (the whole "she is going to die thing"), but apart from the wound nurse, I was left to deal with the fallout of myself. I couldn’t do what used to, and my eating disorder and depression got worse.* (Female, 52yrs, NQ418) |
| Failures in Communication | - No “debrief” - Poor interprofessional communication - Communication of follow up investigations | *I would have liked an explanation about my surgery. What exactly was done or what to expect after. There was a lack of clarify over what happened. I was given a lot of information before the surgery and when on medication so was high and don't remember what was said.* (Female, 77yrs, NQ264)  *I was never told if the operation was successful and if all cancer was gone. An explanation why everything went wrong with the operations I had* (Female, 81yrs, NQ961)  *The GP and district nurse did not know what had happened, and were not qualified to give advice or understand my operation and the aftercare required* (Female, 68yrs, NQ293)  *The continuity of care that is received whilst in hospital does not continue post discharge. There were often communication issues/system/GP issues between services (scans/results/bloods) and no one bothered to tell me the result of my MRI.* (Female, 48yrs, NQ765) |
| Feelings of abandonment and dismissal | - Lack of follow up - Significant delays to corrective surgery - Poor administration support - Difficulties accessing primary and secondary care | *After being discharged, I had no after appointments or follow up from operation, which was very disappointing after major surgery.* (Male, 57yrs, NQ481)  *There was no aftercare whatsoever. The consultant came around once - I couldn’t take it in - he just said, "You've got a stoma - make sure you don't lift anything heavy”. I came home and was "just left to get on with it". I applied for physio - but didn't get a phone call for 3 months later. There was no proper care from GP either. I had to fight to get my stoma reversed.* (Male, 61yrs, NQ4)  *I didn’t answer straight away because I was so angry about being left like this, I am grateful for saving my life but to leave me like this is unacceptable* (Female, 65yrs, NQ57)  *I rang and spoke to secretaries regarding follow up letters and appointments on various occasions. No calls were returned, and letters weren’t received.* (Female, 57yrs, NQ592)  *Every time I ask for help - no one wants to help. It feels like "we saved your life - we don't want anything more to do with it, you’re not our problem anymore”.* (Female, 60yrs, NQ25) |
| Lack of confidence and frustration in services | - Incoherent and conflicting professional advice - Perceptions of misdiagnosis/ missed opportunities - Seeking private sector alternatives | *I was told I would get a follow up letter in the post – I didn’t. I was told I would get an endoscopy date – I didn’t. I was told the stoma nurse would come the following week – she didn’t. I rang the hospital and was told she would come the following week and she still didn’t! I never had an apology. This only heightened my anxiety. (Female, 65yrs, NQ603)*  *I need to see someone as my abdomen seems worse than it was before surgery but hold little hope of seeing anyone because of the COVID situation and because it's not an emergency!* (Female, 69yrs, NQ638)  *I couldn't get an appointment at the doctors! I couldn't get out of bed and fortunately a family friends queued up for me to get an appointment and then the doctor sent me straight in. the next thing I remember was being told "I was to have an operation". I got better I complained to the GP, and they said, "you were lucky". I told them that it was no thanks to them. They said – “you should have said it was an emergency" and I said, "I didn't know it was an emergency". It was a bit late, like closing the stable door after the horse has bolted.* (Male, 89yrs, NQ44)  *I ultimately feel that if I was able to be operated on sooner, I wouldn't have spent another 10 days in hospital followed by another operation.* (Female, 37yrs, NQ925)  *I don't think I ever went back to the doctors after that. It was very frightening, and they didn't listen. I chose to have private follow up as I was fed up with NHS care.* (Female, 54yrs, NQ56) |
